# Subacute changes in brain functional network connectivity after nocturnal sodium oxybate intake are associated with anterior cingulate GABA

**DOI:** 10.1093/cercor/bhad097

**Published:** 2023-03-25

**Authors:** Francesco Bavato, Fabrizio Esposito, Dario A Dornbierer, Niklaus Zölch, Boris B Quednow, Philipp Staempfli, Hans-Peter Landolt, Erich Seifritz, Oliver G Bosch

**Affiliations:** Department of Psychiatry, Psychotherapy and Psychosomatics, Psychiatric University Hospital Zurich, University of Zurich, Zurich 8001, Switzerland; Department of Advanced Medical and Surgical Sciences, University of Campania “Luigi Vanvitelli”, Caserta 81100, Italy; Department of Psychiatry, Psychotherapy and Psychosomatics, Psychiatric University Hospital Zurich, University of Zurich, Zurich 8001, Switzerland; Chronobiology and Sleep Research, Institute of Pharmacology and Toxicology, University of Zurich, Zurich 8001, Switzerland; Department of Psychiatry, Psychotherapy and Psychosomatics, Psychiatric University Hospital Zurich, University of Zurich, Zurich 8001, Switzerland; Department of Forensic Medicine and Imaging, Institute of Forensic Medicine, University of Zurich, Zurich 8001, Switzerland; Department of Psychiatry, Psychotherapy and Psychosomatics, Psychiatric University Hospital Zurich, University of Zurich, Zurich 8001, Switzerland; Neuroscience Center Zurich, University of Zurich and Swiss Federal Institute of Technology Zurich, Zurich 8001, Switzerland; Department of Psychiatry, Psychotherapy and Psychosomatics, Psychiatric University Hospital Zurich, University of Zurich, Zurich 8001, Switzerland; Chronobiology and Sleep Research, Institute of Pharmacology and Toxicology, University of Zurich, Zurich 8001, Switzerland; Department of Psychiatry, Psychotherapy and Psychosomatics, Psychiatric University Hospital Zurich, University of Zurich, Zurich 8001, Switzerland; Neuroscience Center Zurich, University of Zurich and Swiss Federal Institute of Technology Zurich, Zurich 8001, Switzerland; Department of Psychiatry, Psychotherapy and Psychosomatics, Psychiatric University Hospital Zurich, University of Zurich, Zurich 8001, Switzerland

**Keywords:** GHB, GABA, glutamate, salience network, functional connectivity

## Abstract

Sodium oxybate (γ-hydroxybutyrate, GHB) is an endogenous GHB/GABA_B_ receptor agonist, clinically used to promote slow-wave sleep and reduce next-day sleepiness in disorders such as narcolepsy and fibromyalgia. The neurobiological signature of these unique therapeutic effects remains elusive. Promising current neuropsychopharmacological approaches to understand the neural underpinnings of specific drug effects address cerebral resting-state functional connectivity (rsFC) patterns and neurometabolic alterations. Hence, we performed a placebo-controlled, double-blind, randomized, cross-over pharmacological magnetic resonance imaging study with a nocturnal administration of GHB, combined with magnetic resonance spectroscopy of GABA and glutamate in the anterior cingulate cortex (ACC). In sum, 16 healthy male volunteers received 50 mg/kg GHB p.o. or placebo at 02:30 a.m. to maximize deep sleep enhancement and multi-modal brain imaging was performed at 09:00 a.m. of the following morning. Independent component analysis of whole-brain rsFC revealed a significant increase of rsFC between the salience network (SN) and the right central executive network (rCEN) after GHB intake compared with placebo. This SN-rCEN coupling was significantly associated with changes in GABA levels in the ACC (*p*_all_ < 0.05). The observed neural pattern is compatible with a functional switch to a more extrinsic brain state, which may serve as a neurobiological signature of the wake-promoting effects of GHB.

## Introduction

Sodium oxybate (γ-hydroxybutyrate, GHB) is an endogenous GHB/GABA_B_ receptor agonist, which regulates important homeostatic functions such as eating and sexual behavior, as well as the sleep–wake cycle (Liechti et al. 2016). The latter effect is clinically used in narcolepsy and fibromyalgia, where GHB strongly enhances nocturnal slow-wave sleep (Scharf et al. 2003; Plazzi et al. 2014) while promoting next-day wakefulness (Scharf et al. 2003; Black and Houghton 2006). Potential clinical applications of GHB to treat excessive daytime sleepiness have been suggested in different neuropsychiatric disorders including Parkinson disease and depression (Bosch et al. 2012; Buchele et al. 2018). From a neurobiological perspective, these peculiar and complex clinical patterns are not yet fully understood.

Recent neuropsychopharmacological approaches involve the assessment of cerebral resting-state functional connectivity (rsFC) to understand how psychoactive substances differentially modulate brain functioning. Here, an established model to analyze rsFC on a large-scale level involves three core neurocognitive networks: the default mode network (DMN), the central executive network (CEN), and the salience network (SN; Menon 2011). The DMN is located in the posterior cingulate cortex, the medial prefrontal cortex, and the inferior parietal lobule and has an active role for internally directed mental experiences (Mak et al. 2017). The CEN is anchored in the dorsolateral prefrontal cortex and inferior parietal lobule and is mostly involved in goal-directed cognitive operations, such as decision making, problem solving, and in actively maintaining and processing working memory (Pietrzykowski et al. 2022). Finally, the SN has a modulatory function in both rest and task-related activity. It responds to the degree of subjective salience of a stimulus, to control the activity of DMN and CEN, and thereby regulating the switch into different modalities of brain functioning (Goulden et al. 2014; Bolton et al. 2020). Anatomically, the main hubs of SN are the anterior insula and the dorsal part of anterior cingulate cortex (ACC; Menon and Uddin 2010).

Sedative drugs such as midazolam and propofol were shown to reduce DMN and DMN-CEN connectivity (Greicius et al. 2008; Jordan et al. 2013; Liang et al. 2015; Wang et al. 2021), whereas stimulant drugs such as cocaine and modafinil were reported to predominantly enhance CEN and CEN-SN connectivity (Kufahl et al. 2005; Esposito et al. 2013; Cera et al. 2014; Yoo et al. 2018). Under acute GHB challenge with moderate doses, a previous study revealed an acute increase of the SN-DMN and SN-CEN inter-network rsFC, the latter via a regional seed in the mPFC (dorsal nexus) (Bosch et al. 2018). A selective increase of cerebral perfusion in the right anterior insula and in the ACC was also observed in this study, suggesting a crucial role of SN in mediating the acute effects of GHB administration. To date, no study has investigated the post-acute fMRI signal patterns after nocturnal GHB.

In contrast to the growing knowledge of their functional properties, the neurochemical regulation of large-scale brain networks remains largely unknown. Studies applying combined magnetic resonance spectroscopy (MRS) and fMRI reported significant associations between the spectral signals of main inhibitory and excitatory neurotransmitters, gamma-aminobutyric acid (GABA) and glutamate (Glu), and FC, suggesting their role in synchronizing brain activity across specific regions (Harris et al. 2013; Hu et al. 2013; Duncan et al. 2014; Kiemes et al. 2021; Overbeek et al. 2021; Li et al. 2022). This interaction finds a convincing explanation on a neuronal microcircuit level (Logothetis 2008; Kapogiannis et al. 2013), with glutamatergic projection neurons directly activating regional oxygen consumption and blood-oxygen level dependent (BOLD) signal generation (Magistretti and Pellerin 1999; Raichle and Mintun 2006), whereas GABAergic interneurons seem to indirectly reduce BOLD signal activation via inhibitory feedback signaling on glutamatergic transmission (Buzsáki et al. 2007). However, the relevance of this excitatory/inhibitory paradigm for the global modulation of brain activity on a network level remains poorly understood. Some consistent findings suggest local GABA concentrations in main DMN hubs to be negatively associated with intrinsic DMN rsFC and to guide task-related DMN deactivation (Hu et al. 2013; Kapogiannis et al. 2013; Duncan et al. 2014; Chen et al. 2019). In contrast, GABA and Glu concentrations in the SN were reported to be associated with FC in distant brain regions rather than controlling activity in the SN itself (Harris et al. 2013; Wang et al. 2017; Levar et al. 2019; Overbeek et al. 2021). However, most studies were focused on specific interactions between single network nodes rather than global modulation of network activity (Duncan et al. 2014; Kiemes et al. 2021) and pharmacological studies combining rsFC and neurometabolic investigations in humans are still limited (Egerton 2021).

To elucidate the neural underpinnings of the above described wake-promoting effects of GHB, we investigated whole-brain rsFC of the DMN, the CEN, and the SN, combined with GABA and Glu levels in the ACC in the morning after nocturnal application of the drug in 16 healthy male volunteers using a placebo-controlled, double-blind, randomized, cross-over pharmacological fMRI design. The MRS-seed in the ACC was selected as recent studies demonstrated that neurochemical balance in this brain region modulate network interconnectivity (Levar et al. 2019), and the involvement of the ACC was consistently reported in acute challenges with GHB (Bosch et al. 2017a, 2018; von Rotz et al. 2017; Dornbierer et al. 2019a). We hypothesized that GHB would switch the rsFC configuration into an extrinsic CEN-directed brain state, coherently with its known post-acute wakefulness-enhancing effects. We also expected a significant interaction of altered Glu and GABA levels in the ACC with global changes of rsFC patterns, thus supporting a crucial role of neurochemical balance in this brain area in modulating neural effects by GHB.

## Methods

### Permission

The study was approved by the Swiss Agency for Therapeutic Products (Swissmedic) as well as by the Ethics Committee of the Canton of Zurich and registered at ClinicalTrials.gov (NCT02342366). All participants provided written informed consent according to the declaration of Helsinki.

### Study design

The study followed a randomized, placebo-controlled, order-balanced, double-blind, cross-over design. Two experimental nights (GHB vs. placebo) were separated by a washout phase of 7 days. Prior to definitive enrollment into the study, all participants underwent a polysomnographic examination in the sleep laboratory of the Institute of Pharmacology and Toxicology of the University of Zurich to exclude sleep-related disorders such as sleep apnea, restless legs syndrome, sleep onset rapid-eye movement sleep and reduced sleep efficiency (< 80%). To allow for habituation to the sleep laboratory setting, each experimental night was preceded by an adaptation night. Apart from the here presented post-sleep fMRI resting-state networks (RSN) results, GHB effects on sleep neurophysiology (Dornbierer et al. 2019a), kynurenine pathway metabolites (Dornbierer et al. 2019b), and post-awakening brain metabolite signals and vigilance (Dornbierer et al. 2023) were also assessed in the experiment and published elsewhere.

### Participants

In sum, 20 healthy, male volunteers completed the study, whereof 4 participants were excluded from the final data analysis due to technical issues with the MR scanner or insufficient MR data quality (mean age of included participants: 25.8 ± 5.1 years). The following criteria were required for inclusion: (i) male sex (to avoid potential impact of menstrual cycle on primary outcome variables (De Bondt et al. 2015); (ii) age within the range of 18 to 40 years; (iii) absence of somatic, neurologic, and psychiatric disorders; (iv) no first-degree relatives with a history of highly heritable psychiatric disorders such as schizophrenia, bipolar disorder, autism, and attention-deficit/hyperactivity disorder (ADHD); (v) non-smoker; (vi) no history of regular drug use (lifetime use < 5 occasions of each drug, except occasional cannabis use). No participant reported previous experiences with GHB in their life. Participants had to refrain from illegal drugs for two weeks and from caffeine for one week prior to the first experimental night and throughout the entire study. No use of alcohol was allowed 24 h before each study night. Participants were instructed to keep a regular sleep–wake rhythm with 8-h time-in-bed from 23:00 p.m. to 07:00 a.m. during one week prior to the first experimental night and in the week between the two experimental nights. To ensure compliance with this requirement, participants wore an actimeter on the non-dominant arm and kept a sleep–wake diary. All volunteers received a monetary compensation for their study participation. The initial sample size was selected according to power analysis showing that, given a power of 90%, α = 5%, and *n* = 20, medium to large effects (*f* = 0.38 for within-subject effects) can be reliably detected. Previous studies with similar sample sizes investigating acute and subacute effects of GHB administration on sleep and day-after wakefulness reported large effect sizes (Bosch et al. 2018; Dornbierer et al. 2019a, 2023).

### Urine immunoassay

Urine samples were taken on each test night, to ensure abstinence from illegal drug use (Drug-Screen Multi 12-AE, Nal von Minden GmbH, Regensburg, DE).

### Drug administration

**Fig. 1:**
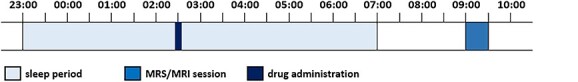
Study design of the experimental nights. Sleep period (23:00–07:00), time point of drug administration (02:30) and MRS/MRI scan (09:00) are indicated.

At each experimental night, study participants were awoken at 02:30 a.m. to receive 50 mg/kg of GHB (Xyrem®) or placebo dissolved in 2 dL of orange juice, matched in appearance and taste (see Fig. 1). This dose represents the maximal therapeutic starting dose in narcolepsy. After GHB/placebo intake, volunteers where allowed to immediately return to sleep. GHB administration in the middle of the night was chosen because of its short half-lifetime and its potency to enhance deep sleep in the second half of the night, after the physiological dissipation of sleep intensity and propensity (Dornbierer et al. 2019a).

### MRI data acquisition

The fMRI resting state scan was performed in the morning after both experimental nights on a Philips Achieva 3T whole-body MR-unit equipped with a 32-channel head coil (Philips Medical Systems, Best, The Netherlands). The session started at 09:00 a.m. with a T1-weighted anatomical brain scan and was followed by fMRI acquisition (5- and 10-min duration, respectively). RsFC time series were acquired with a sensitivity-encoded single-shot echo-planar imaging sequence (SENSE-sshEPI). The rsFC protocol used the following acquisition parameters: TE = 35 ms, TR = 3,000 ms, flip angle = 82°, FOV = 220 mm, acquisition matrix = 80 × 80 (in plane voxel size = 2.75 × 2.75 mm^2^), 32 contiguous axial slices (placed along the anterior–posterior commissure plane) with a slice thickness of 4 mm and a SENSE factor *R*=2.0. For structural reference, a magnetization prepared rapid gradient-echo (MP-RAGE) T1-weighted anatomical scan was acquired with the following parameters: TR/TE = 9.3/4.6 ms, flip angle = 8°, 160 sagittal slices, FOV = 240 × 240 mm^2^, voxel size = 1 × 1 × 1 mm^3^. A single voxel (sv-) MRS was performed at each experimental session immediately before the fMRI paradigm, using two-dimensional J-resolved spectroscopy combined with PRESS localization (2D-JPRESS) sequence. The voxel with an effective size of 14.8 cm^3^ was placed in the anterodorsal portion of the ACC (see MRS-protocol details in a separate publication and in Supplementary Material; Dornbierer et al. 2023). The minimal TE of 30 ms was incremented in 100 steps of 2 ms. The TR was set at 1,600 ms and we acquired eight averages for each TE-step. Metabolites are calculated as ratios to the total creatine.

### MRI data preprocessing

Standard image data preparation and pre-processing as well as statistical analysis and visualization were performed in Matlab (The Mathworks Inc., United States) and BrainVoyager (Brain Innovation B.V., The Netherlands). Functional data preprocessing included a correction for slice scan timing acquisition, a 3D rigid body motion correction, a spatial smoothing (Gaussian kernel of 6-mm full-width at half-maximum), a temporal high-pass filter with cut-off set to 0.0080 Hz per time-course and a temporal low-pass filter (Gaussian kernel of 3 s). The mean frame wise displacement was estimated from each time-series prior to nuisance and motion regression to exclude motion-driven bias in connectivity correlations (Power et al. 2012). For further details, see Supplementary Material and previous publications (Bosch et al. 2017b, 2018).

### Statistical analysis of fMRI images

#### Independent component analysis of resting-state fMRI networks

The independent component analysis (ICA) analysis of RSN networks followed the identical approach described in a previous study of ours (Bosch et al. 2018). The analysis is based on a hierarchical approach specifically designed to study FC under changing experimental conditions (see also (Esposito et al. 2014)) in which first- (single-subject, single-scan) and second-level (group) analyses are performed using the fastICA (Hyvarinen 1999) and the self-organizing group-level ICA algorithm (Esposito et al. 2005). For each scan condition (GHB vs. placebo), 30 ICA components were extracted using fastICA, roughly corresponding to 1/6 of the number of time points (see also Greicius et al. 2004; Shirer et al. 2015). A more detailed description is available in Supplementary Material and previous publications (Bosch et al. 2018).

#### Analysis of correlations between RSN-FC and single voxel MRS metabolites

To explore possible interactions between neurochemical brain balance and RSN-FC, we also investigated associations of FC alterations between conditions with the respective changes of metabolite signals measured by single voxel sv-MRS analysis. The change in metabolite spectral signals across experimental conditions was calculated subtracting the metabolite signlas of the GHB condition from signals of placebo condition (e.g. [ΔGABA] = [GABA at placebo condition] – [GABA at GHB condition]).

For internetwork connectivity, the change of correlation-*z*-values (ΔrsFC) across conditions in the internetwork connectivity matrices was calculated subtracting the z-value of GHB condition from z-value of placebo condition ([ΔrsFC] = [*z*-value at placebo session] – [*z*-value at GHB session]) for all network-pairs showing significant changes of between-network connectivity in the paired *t*-test of connectivity matrices (*P* < 0.05). Finally, generalized linear models with normal distribution and identity link function were performed to assess associations between ΔrsFC for significantly altered internetwork connections and metabolites changes expressed as ΔGABA and ΔGlu, after controlling for experimental session order (GHB first vs. placebo first).

#### Subjective state variables

Each participant’s post-awakening mental state was assessed at 10:00 a.m. using the self-report questionnaire EWL-60 (“Eigenschaftswörterliste”; Janke and Debus 1986). The EWL-60 is an established rating scale assessing multidimensional aspects of subjective mental state, which has been found to be well suited to measure short-term changes induced by psychoactive drugs (Studerus et al. 2010). It is composed of a list of 60 adjectives (e.g. “active,” “sorrowful,” “tired,” “sociable”) that participants have to rate on four-point Likert scales ranging from 0 (not at all) to 3 (very much). Single item scores can be grouped into six subscales (i.e. performance-related activation, general inactivation, extro/introversion, general well-being, emotional sensitivity, depressiveness/anxiety). Generalized linear models with normal distribution and identity link function were performed to assess associations between EWL subscale scores and condition (GHB vs. placebo), after controlling for experimental session order. All statistical tests were carried out at a significance level of *P* < 0.05 and were performed using SPSS 26.0, R-Studio, and JASP.

## Results

### fMRI data and RSNs

**Fig. 2:**
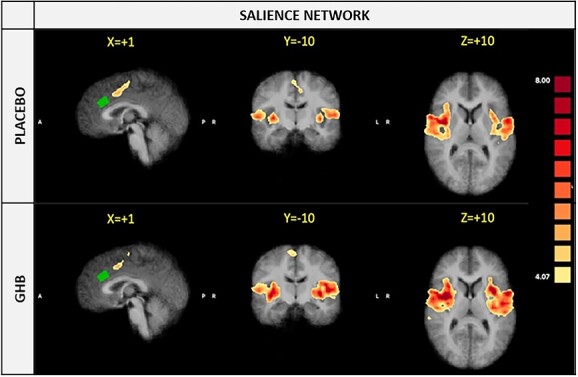
Spatial distribution of bilateral SN in the ICA at both placebo and GHB conditions. The SN is shown in three planes on average Talairach anatomical scan. The MRS-voxel is shown in all planes (green). After correcting for all voxel-level comparisons, no compact clusters with a statistically significant effect was detected (all *P* < 0.05).

**Fig. 3:**
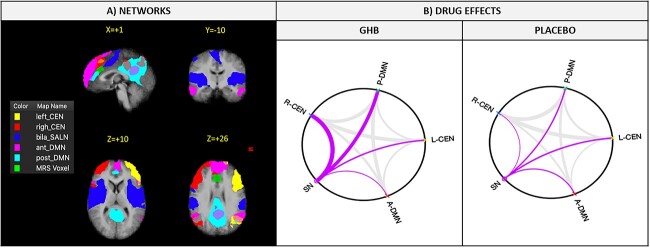
RSNs and internetwork connectivity patterns. (A) All RSNs are shown in three planes on average Talairach anatomical scan. (B) The anterior and posterior default mode network (A-DMN, P-DMN), the L-CEN, R-CEN, and the SN are considered. The network graph highlights the connections of the SN with the other RSNs (B).

**Fig. 4:**
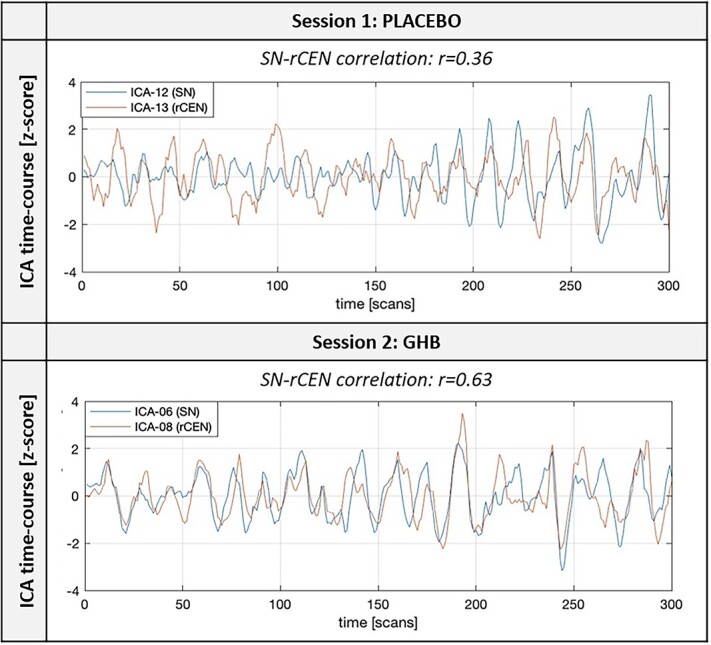
Time-course of functional connectivity in two components from the ICA corresponding to the SN and the rCEN, obtained from a single subject at both placebo and GHB conditions. The correlation coefficient was calculated between the time-courses of the two networks. The increase in the dynamic coupling of SN-rCEN is testified by a correlation coefficient changing from *r* = 0.36 to 0.63.

For both conditions (GHB and placebo), the mean frame-wise displacement was below the critical threshold of 0.5 mm and did not differ between the conditions. Using the network template masks for extracting the homolog network best-fitting ICA components from each subject, we examined the differences between conditions (GHB vs. placebo), in both within- (via voxel-wise analysis) and between-network (via correlation analysis) connectivity. No significant drug effects were found within the DMN, the CEN, and the SN (see Fig. 2). In the internetwork functional connectivity analysis, we observed a significant effect in the internetwork connectivity of the right central executive network (rCEN) with the SN in the GHB condition (one sample *t*-test: *P* = 0.038), which was not present under placebo (one sample *t*-test: *P* = 0.460). Coherently, rCEN-SN internetwork connectivity was significantly higher in the GHB condition compared with placebo (GHB vs. placebo, paired *t*-test: *P* = 0.017, Cohens’*d* = 0.49) (see Fig. 3 and Table 1). The time-course of the rCEN-SN coupling in a representative study participant is visualized in Fig. 4 (further detailed examples from single participants are available in the Supplementary Material).

**Table 1:** Internetwork connectivity scores.

**Networks**	**Placebo (*z*-score)**	**GHB (*z*-score)**	**Statistics**
Left CEN—anterior DMN	0.04 ± 0.28	0.04 ± 0.26	*T* = −0.01, *P* = 0.49
Right CEN—anterior DMN	0.05 ± 0.25	0.06 ± 0.21	*T* = **−**0.84, *P* = 0.47
Left CEN—right CEN	0.12 ± 0.22	0.10 ± 0.22	*T* = 0.37, *P* = 0.36
Posterior DMN—anterior DMN	0.07 ± 0.25	0.08 ± 0.22	*T* = **−**0.13, *P* = 0.45
Posterior DMN—left CEN	0.06 ± 0.19	0.04 ± 0.22	*T* = 0.26, *P* = 0.40
Posterior DMN—right CEN	0.12 ± 0.29	0.09 ± 0.24	*T* = 0.41, *P* = 0.34
SN—left CEN	0.04 ± 0.24	0.03 ± 0.30	*T* = 0.09, *P* = 0.46
**SN—right CEN**	**−0.01 ± 0.22**	**0.10 ± 0.21**	*T* = **−2.32, *P* = 0.017**
SN—anterior DMN	0.02 ± 0.28	**−**0.01 ± 0.25	*T* = 0.43, *P* = 0.34
SN—posterior DMN	0.03 ± 0.23	0.08 ± 0.26	*T* = **−**0.86, *P* = 0.20

The table reports means ± standard deviations. Significant group differences are shown in bold. The internetwork connectivity is quantified using a correlation *z*-value calculated for each network-pair. *T*: Student *t*-test; CEN: central executive network; DMN: default mode network; SN: salience network.

### Associations between RSN-rsFC and sv-MRS metabolite signals

**Fig. 5:**
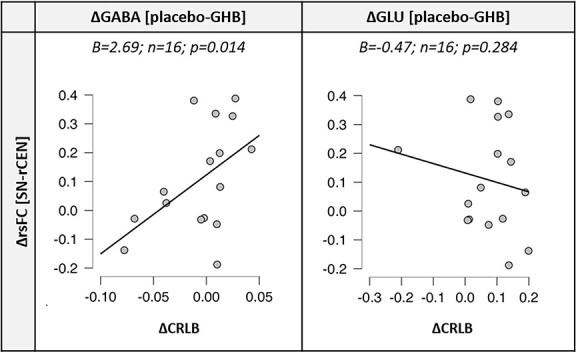
Scatterplots showing associations between the GHB-induced changes in resting-state functional connectivity between the SN and rCEN connection (ΔrsFC SN-rCEN) and the GHB-induced changes of metabolite signals in the ACC (ΔGABA, ΔGlu). In the *y*-axis, the change of rsFC is obtained by extracting the correlation-*z*-values in the internetwork connectivity matrices. The changes of metabolite signals in the *x*-axis are reported as mean differences of relative Cramér–Rao lower bounds (ΔCRLB). Generalized linear models with normal distribution and identity link function were performed.

MRS data have been reported in detail in a separate publication (Dornbierer et al. 2023). Generalized linear regression models revealed that ΔrsFC of the rCEN-SN was significantly associated with ΔGABA (B = 2.69; SE = 1.10; 95% CI: 0.54, 4.84; *P* = 0.014) but not with ΔGlu (B = -0.47; SE = 0.44; 95% CI: −1.33, 0.39; *P* = 0.284), after correction for experimental session order (see Fig. 5).

### Subjective drug effects

Generalized linear regression models revealed no significant effects of condition (GHB vs. placebo) and experimental session order on morning EWL subscale scores (all *P* > 0.13, see Supplementary Material).

## Discussion

The present study aimed at investigating the neuropsychopharmacological effects of a nocturnal dose of 50 mg/kg GHB p.o. on next mornings’ rsFC and its relationship to GABA and Glu alterations in the ACC. First, we observed a newly induced rCEN-SN coupling after a night with GHB, which was not present in the placebo condition; second, we found that this rsFC alteration was significantly associated with GABA changes in the ACC.

In previous studies, GHB was found to acutely modulate rsFC patterns. In particular, GHB administration (35 mg/kg p.o.) in wake healthy individuals acutely increased rsFC between the DMN and the SN (at 34 min after GHB-intake) compared with placebo, without significantly affecting the spatial rsFC distribution of all major large-scale networks (Bosch et al. 2018). Consistently, the here reported post-acute alterations across the same large-scale networks did not affect the within-network rsFC distributions, and the observed switching from DMN-SN (acute, previous studies) (Bosch et al. 2018) to rCEN-SN (post-acute, current study) coupling confirms the SN being a critical target of GHB. In line with this hypothesis, a local increase in cerebral blood flow under acute GHB was also detected in main hubs of the SN, such as the ACC and the right anterior insula, which were correlated with increased relaxation, and body/emotion awareness (Bosch et al. 2017a). Similarly, current source density EEG analysis revealed significant spectral alterations in the ACC under acute challenge with 35 (wake condition; von Rotz et al. 2017) and 50 mg/kg GHB p.o. (sleep condition) in two separate observations (Dornbierer et al. 2019a). Thus, activation of the SN, and thereby modulation of the balance between DMN and CEN is a core neuronal effect of GHB at both acute and post-acute phases.

These findings are particularly intriguing when considering the detailed functional meaning of the SN and the unique psychopharmacological and behavioral profile of GHB. The SN has been proposed as a detector of saliency to guide adaptive behavior (Goulden et al. 2014). Anchored in the anterior insula, the dorsal ACC, as well as other subcortical structures, the SN responds to biologically relevant internal and external stimuli and modulates the switch into different brain states by regulation of CEN/DMN activity. Accordingly, SN dysfunctions were frequently associated with different symptom dimensions in neuropsychiatric disorders. While increased rsFC between main SN and DMN hubs was consistently related to depressive symptoms and ruminations (Brakowski et al. 2017; Zhou et al. 2020), intrinsic alterations in the SN and disturbed CEN-DMN transition were linked to reality distortion and cognitive deficits in schizophrenia spectrum disorder (Bolton et al. 2020).

Now, translating these findings to GHB neuropsychopharmacology, one may expect to observe different network patterns coherently to the different behavioral effects of GHB in the acute and post-acute phase. In the acute phase, 35 mg/kg oral GHB-induced mixed sedative/stimulant behavioral effects, which were accompanied by increased SN-DMN connectivity (Bosch et al. 2018). This rsFC pattern indicates a switch into a more internally directed brain state, compatible with reduced attentional focus to external stimuli (Doll et al. 2015; Bosch and Seifritz 2016; Liechti et al. 2016; Liakoni et al. 2018). Similar rsFC patterns were also observed during mindfulness, light propofol sedation, and increased homeostatic sleep pressure after sleep deprivation (Bosch et al. 2013; Guldenmund et al. 2013; Doll et al. 2015). In contrast, the post-acute behavioral effects of nocturnal 50 mg/kg oral GHB included activation and vigilance enhancement (Robinson and Keating 2007; Buchele et al. 2018; Dornbierer et al. 2023). Thus, the increased rCEN-SN rsFC we observed in this study is coherent with the previously reported stimulant effects of the substance and suggests a post-acute switch into a more externally directed state of brain functioning. In line with that notion, increased rsFC between hubs of the CEN and SN was already related to enhanced attentional focus in meditation practioners at rest (Hasenkamp and Barsalou 2012) and to symptoms improvement in children with ADHD treated with methylphenidate (Yoo et al. 2018). Moreover, reduced rsFC between main hubs of the CEN and SN was frequently associated with fatigue and daytime sleepiness in disorders such as narcolepsy, depression, chronic fatigue syndrome, fibromyalgia, and Parkinson disease (Ichesco et al. 2014; Pannekoek et al. 2014; Gay et al. 2016; Xiao et al. 2019). Thus, increased CEN-SN coupling could represent a neural signature of stimulant effects of GHB and offer a clinical marker for its therapeutic use. Importantly, we did not find any significant effects of GHB on the subjective mental state in this study. However, we observed increased sustained vigilant attention (assessed with a 10-min visual psychomotor vigilance test), which was tested in the same experiment and reported in a previous publication (Dornbierer et al. 2023).

As a note, the increase of rsFC between the SN and the rCEN, but not the lCEN, is coherent with previous findings showing asymmetric effects of GHB on RSNs. Increased cerebral blood flow in the right anterior insula and increased rSN-rCEN coupling via the dorsal nexus have been reported after acute GHB challenge (Bosch et al. 2017a, 2018). This lateralization may be driven by the intrinsic organization of SN, as right hubs are considered crucial in regulating the interaction with the CEN (Menon 2015). A physiological lateralization of the CEN has also been postulated in the past, with a right-dominance described for visuospatial attention and response inhibition (Kaller et al. 2010). In contrast, a recent meta-analysis reported no evidence for the lateralization of cognitive domains related to attention (Karolis et al. 2019). Pharmaco-imaging studies using stimulant substances have so far shown inconsistent findings regarding the lateralized involvement of the CEN (Kufahl et al. 2005; Esposito et al. 2013; Cera et al. 2014).

To investigate how the reported modulation of rsFC alterations relate to the neurochemical brain homeostasis, we analyzed previously published data of MRS from an ACC-seed assessed in the same individuals and from the same experiment (Dornbierer et al. 2023). The selection of the ACC-seed to study rsFC-regulation was driven by previous evidence showing that the ACC is a crucial target of GHB effects (Bosch et al. 2017a, 2018; von Rotz et al. 2017; Dornbierer et al. 2019a, 2023) and numerous reports on the ACCs’ role in controlling global brain function (Chen et al. 2019; Levar et al. 2019). In the current analysis, ΔGABA but not ΔGlu levels predicted the increased rCEN-SN coupling. These findings, are coherent with other reports in the literature, which described cingulate GABA to be positively associated with DMN deactivation and increased CEN connectivity (Hu et al. 2013; Kapogiannis et al. 2013; Levar et al. 2019). In particular, Levar and colleagues recently demonstrated GABA levels in the ACC to be positively correlated with rsFC in the left and right CEN while being negatively correlated to rsFC between CEN and DMN (Levar et al. 2019). The authors also described a negative association of GABA/Glx (with Glx considered as glutamine + Glu) levels with rsFC in the left insula and left occipital cortex but no correlations between Glx levels and rsFC. Notably, despite the ACC being itself part of the SN, GABA levels in this area were positively correlated with rsFC in the CEN but not in the SN, supporting the view of a neurochemical balance in the ACC to be involved in functional control of distant brain areas. Therefore, our findings provide an experimental validation of the role of GABA in controlling large-scale rsFC, which has been previously reported in cross-sectional investigations in healthy individuals and in patients with psychiatric disorders, but has been barely addressed in pharmacological studies (Egerton 2021; Kiemes et al. 2021).

Our study bears several limitations. The final sample size of *n* = 16 resulted in lower statistical power than initially planned, thus limiting the generalizability of our findings. Nonetheless, medium to large effect sizes could still be detected. MRS provides the total metabolite signals in a given ROI, which is why different metabolite pools (e.g. cytoplasmic, vesicular, or extracellular) cannot be differentiated. Thus, it remains unclear if the observed alterations of Glu are related to increased glutamatergic transmission in the ACC or to other indirect metabolic alterations in the region (Dornbierer et al. 2023). Obviously, MRS-data also limit our investigation to the ACC and we cannot exclude concomitant neurochemical modulation of rsFC through other pathways. As such, similar neurochemical alterations could also occur in other crucial SN hubs such as the anterior insula. Moreover, the relevance of mesolimbic dopamine levels for SN regulation was already described in the literature and may also contribute to the effects of GHB on rsFC (Bosch and Seifritz 2016; McCutcheon et al. 2019). In addition, our study sample consisted solely of young healthy men, to avoid the possible interference of hormonal fluctuations throughout the menstrual cycle on brain metabolite levels (De Bondt et al. 2015). This, taken together with the small sample size, does not yet allow to generalize our results to the entire healthy population.

In conclusion, the present study provided evidence of persisting internetwork connectivity changes in the morning following a nocturnal therapeutic dose of GHB in humans. The observed alteration in connectivity pattern seem to indicate a modulation of the balancing function of the SN between the DMN and the CEN, toward a more externally oriented brain state, which is in line with GHB’s ability to improve next-day waking functions. We also described a GHB-induced positive interaction of GABA/Glu balance in the ACC with whole-brain connectivity changes. Thus, our findings support the idea of an excitatory/inhibitory equilibrium in the ACC to be actively involved in the modulation of rsFC on a large-scale level. Future research should clarify the generalizability of these findings to other stimulant and/or sedative drugs affecting GABA and Glu homeostasis and further assess correlations with cognitive and behavioral effects in clinical populations.

## Supplementary material

Supplementary material is available at *Cerebral Cortex* online.

## Supplementary Material

Bavato_et_al_2023_Suplemetary_materials_final_bhad097

## Data Availability

Anonymized data will be shared by request with any qualified investigator with institutional review board approval for the purposes of validation and/or replication using our center’s established procedures for sharing data. *Conflict of Interest statement.* None declared.

## References

[ref1] Black J, Houghton WC. Sodium oxybate improves excessive daytime sleepiness in narcolepsy. Sleep. 2006:29:939–946. 16895262 10.1093/sleep/29.7.939

[ref2] Bolton TAW, Wotruba D, Buechler R, Theodoridou A, Michels L, Kollias S, Rossler W, Heekeren K, Van De Ville D. Triple network model dynamically revisited: lower salience network state switching in pre-psychosis. Front Physiol. 2020:11:66. 32116776 10.3389/fphys.2020.00066 PMC7027374

[ref3] Bosch OG, Seifritz E. The behavioural profile of gamma-hydroxybutyrate, gamma-butyrolactone and 1,4-butanediol in humans. Brain Res Bull. 2016:126:47–60. 26855327 10.1016/j.brainresbull.2016.02.002

[ref4] Bosch OG, Quednow BB, Seifritz E, Wetter TC. Reconsidering GHB: orphan drug or new model antidepressant? J Psychopharmacol. 2012:26:618–628. 21926421 10.1177/0269881111421975

[ref5] Bosch OG, Rihm JS, Scheidegger M, Landolt HP, Stampfli P, Brakowski J, Esposito F, Rasch B, Seifritz E. Sleep deprivation increases dorsal nexus connectivity to the dorsolateral prefrontal cortex in humans. Proc Natl Acad Sci USA. 2013:110:19597–19602. 24218598 10.1073/pnas.1317010110 PMC3845164

[ref6] Bosch OG, Esposito F, Havranek MM, Dornbierer D, von Rotz R, Staempfli P, Quednow BB, Seifritz E. Gamma-hydroxybutyrate increases resting-state limbic perfusion and body and emotion awareness in humans. Neuropsychopharmacology. 2017a:42:2141–2151. 28561068 10.1038/npp.2017.110 PMC5603804

[ref7] Bosch OG, Havranek MM, Baumberger A, Preller KH, Von Rotz R, Herdener M, Kraehenmann R, Staempfli P, Scheidegger M, Klucken T, et al. Neural underpinnings of prosexual effects induced by gamma-hydroxybutyrate in healthy male humans. Eur Neuropsychopharmacol. 2017b:27:372–382. 28284776 10.1016/j.euroneuro.2017.02.006

[ref8] Bosch OG, Esposito F, Dornbierer D, Havranek MM, von Rotz R, Kometer M, Staempfli P, Quednow BB, Seifritz E. Gamma-hydroxybutyrate increases brain resting-state functional connectivity of the salience network and dorsal nexus in humans. NeuroImage. 2018:173:448–459. 29524621 10.1016/j.neuroimage.2018.03.011

[ref9] Brakowski J, Spinelli S, Dörig N, Bosch OG, Manoliu A, Holtforth MG, Seifritz E. Resting state brain network function in major depression—depression symptomatology, antidepressant treatment effects, future research. J Psychiatr Res. 2017:92:147–159. 28458140 10.1016/j.jpsychires.2017.04.007

[ref10] Buchele F, Hackius M, Schreglmann SR, Omlor W, Werth E, Maric A, Imbach LL, Hagele-Link S, Waldvogel D, Baumann CR. Sodium oxybate for excessive daytime sleepiness and sleep disturbance in Parkinson disease: a randomized clinical trial. JAMA Neurol. 2018:75:114–118. 29114733 10.1001/jamaneurol.2017.3171 PMC5833497

[ref11] Buzsáki G, Kaila K, Raichle M. Inhibition and brain work. Neuron. 2007:56:771–783. 18054855 10.1016/j.neuron.2007.11.008 PMC2266612

[ref12] Cera N, Tartaro A, Sensi SL. Modafinil alters intrinsic functional connectivity of the right posterior insula: a pharmacological resting state fMRI study. PLoS One. 2014:9:e107145. 25237810 10.1371/journal.pone.0107145 PMC4169531

[ref13] Chen X, Fan X, Hu Y, Zuo C, Whitfield-Gabrieli S, Holt D, Gong Q, Yang Y, Pizzagalli DA, Du F, et al. Regional GABA concentrations modulate inter-network resting-state functional connectivity. Cereb Cortex. 2019:29:1607–1618. 29608677 10.1093/cercor/bhy059 PMC6418388

[ref14] De Bondt T, De Belder F, Vanhevel F, Jacquemyn Y, Parizel PM. Prefrontal GABA concentration changes in women—influence of menstrual cycle phase, hormonal contraceptive use, and correlation with premenstrual symptoms. Brain Res. 2015:1597:129–138. 25481417 10.1016/j.brainres.2014.11.051

[ref15] Doll A, Holzel BK, Boucard CC, Wohlschlager AM, Sorg C. Mindfulness is associated with intrinsic functional connectivity between default mode and salience networks. Front Hum Neurosci. 2015:9:461. 26379526 10.3389/fnhum.2015.00461 PMC4548211

[ref16] Dornbierer DA, Baur DM, Stucky B, Quednow BB, Kraemer T, Seifritz E, Bosch OG, Landolt HP. Neurophysiological signature of gamma-hydroxybutyrate augmented sleep in male healthy volunteers may reflect biomimetic sleep enhancement: a randomized controlled trial. Neuropsychopharmacology. 2019a:44:1985–1993. 30959514 10.1038/s41386-019-0382-z PMC6785068

[ref17] Dornbierer DA, Boxler M, Voegel CD, Stucky B, Steuer AE, Binz TM, Baumgartner MR, Baur DM, Quednow BB, Kraemer T, et al. Nocturnal Gamma-Hydroxybutyrate reduces cortisol-awakening response and morning kynurenine pathway metabolites in healthy volunteers. Int J Neuropsychopharmacol. 2019b:22:631–639. 31504554 10.1093/ijnp/pyz047 PMC6822136

[ref18] Dornbierer DA, Zölch N, Baur DM, Hock A, Stucky B, Quednow BB, Kraemer T, Seifritz E, Bosch OG, Landolt H-P. Nocturnal sodium oxybate increases morning anterior cingulate glutamate signal. J Sleep Res. accepted. 2023:e13866. 10.1111/jsr.13866. 36869598

[ref19] Duncan NW, Wiebking C, Northoff G. Associations of regional GABA and glutamate with intrinsic and extrinsic neural activity in humans—a review of multimodal imaging studies. Neurosci Biobehav Rev. 2014:47:36–52. 25066091 10.1016/j.neubiorev.2014.07.016

[ref20] Egerton A . The potential of 1H-MRS in CNS drug development. Psychopharmacology. 2021:238:1241–1254. 31486875 10.1007/s00213-019-05344-7 PMC8062504

[ref21] Esposito F, Scarabino T, Hyvarinen A, Himberg J, Formisano E, Comani S, Tedeschi G, Goebel R, Seifritz E, Di Salle F. Independent component analysis of fMRI group studies by self-organizing clustering. NeuroImage. 2005:25:193–205. 15734355 10.1016/j.neuroimage.2004.10.042

[ref22] Esposito R, Cilli F, Pieramico V, Ferretti A, Macchia A, Tommasi M, Saggino A, Ciavardelli D, Manna A, Navarra R, et al. Acute effects of modafinil on brain resting state networks in young healthy subjects. PLoS One. 2013:8:e69224. 23935959 10.1371/journal.pone.0069224 PMC3723829

[ref23] Esposito F, Otto T, Zijlstra FR, Goebel R. Spatially distributed effects of mental exhaustion on resting-state FMRI networks. PLoS One. 2014:9:e94222. 24705397 10.1371/journal.pone.0094222 PMC3976406

[ref24] Gay CW, Robinson ME, Lai S, O'Shea A, Craggs JG, Price DD, Staud R. Abnormal resting-state functional connectivity in patients with chronic fatigue syndrome: results of seed and data-driven analyses. Brain Connect. 2016:6:48–56. 26449441 10.1089/brain.2015.0366 PMC4744887

[ref25] Goulden N, Khusnulina A, Davis NJ, Bracewell RM, Bokde AL, McNulty JP, Mullins PG. The salience network is responsible for switching between the default mode network and the central executive network: replication from DCM. NeuroImage. 2014:99:180–190. 24862074 10.1016/j.neuroimage.2014.05.052

[ref26] Greicius MD, Srivastava G, Reiss AL, Menon V. Default-mode network activity distinguishes Alzheimer’s disease from healthy aging: evidence from functional MRI. Proc Natl Acad Sci. 2004:101:4637–4642. 15070770 10.1073/pnas.0308627101 PMC384799

[ref27] Greicius MD, Kiviniemi V, Tervonen O, Vainionpaa V, Alahuhta S, Reiss AL, Menon V. Persistent default-mode network connectivity during light sedation. Hum Brain Mapp. 2008:29:839–847. 18219620 10.1002/hbm.20537 PMC2580760

[ref28] Guldenmund P, Demertzi A, Boveroux P, Boly M, Vanhaudenhuyse A, Bruno MA, Gosseries O, Noirhomme Q, Brichant JF, Bonhomme V, et al. Thalamus, brainstem and salience network connectivity changes during propofol-induced sedation and unconsciousness. Brain Connect. 2013:3:273–285. 23547875 10.1089/brain.2012.0117

[ref29] Harris RE, Napadow V, Huggins JP, Pauer L, Kim J, Hampson J, Sundgren PC, Foerster B, Petrou M, Schmidt-Wilcke T, et al. Pregabalin rectifies aberrant brain chemistry, connectivity, and functional response in chronic pain patients. Anesthesiology. 2013:119:1453–1464. 24343290 10.1097/ALN.0000000000000017

[ref30] Hasenkamp W, Barsalou LW. Effects of meditation experience on functional connectivity of distributed brain networks. Front Hum Neurosci. 2012:6:38. 22403536 10.3389/fnhum.2012.00038 PMC3290768

[ref31] Hu Y, Chen X, Gu H, Yang Y. Resting-state glutamate and GABA concentrations predict task-induced deactivation in the default mode network. J Neurosci. 2013:33:18566–18573. 24259578 10.1523/JNEUROSCI.1973-13.2013 PMC3834059

[ref33] Hyvarinen A . Fast and robust fixed-point algorithms for independent component analysis. IEEE Trans Neural Netw. 1999:10:626–634. 18252563 10.1109/72.761722

[ref34] Ichesco E, Schmidt-Wilcke T, Bhavsar R, Clauw DJ, Peltier SJ, Kim J, Napadow V, Hampson JP, Kairys AE, Williams DA, et al. Altered resting state connectivity of the insular cortex in individuals with fibromyalgia. J Pain. 2014:15:815, e811–826. 24815079 10.1016/j.jpain.2014.04.007 PMC4127388

[ref35] Janke W, Debus G. EWL 60 S. In: CIPS, editor. Internationale Skalen für Psychiatrie. Weinheim, Germany: Beltz. 1986. pp. 43–47.

[ref36] Jordan D, Ilg R, Riedl V, Schorer A, Grimberg S, Neufang S, Omerovic A, Berger S, Untergehrer G, Preibisch C, et al. Simultaneous electroencephalographic and functional magnetic resonance imaging indicate impaired cortical top-down processing in association with anesthetic-induced unconsciousness. Anesthesiology. 2013:119:1031–1042. 23969561 10.1097/ALN.0b013e3182a7ca92

[ref37] Kaller CP, Rahm B, Spreer J, Weiller C, Unterrainer JM. Dissociable contributions of left and right dorsolateral prefrontal cortex in planning. Cereb Cortex. 2010:21:307–317. 20522540 10.1093/cercor/bhq096

[ref38] Kapogiannis D, Reiter DA, Willette AA, Mattson MP. Posteromedial cortex glutamate and GABA predict intrinsic functional connectivity of the default mode network. NeuroImage. 2013:64:112–119. 23000786 10.1016/j.neuroimage.2012.09.029 PMC3801193

[ref39] Karolis VR, Corbetta M, Thiebaut de Schotten M. The architecture of functional lateralisation and its relationship to callosal connectivity in the human brain. Nat Commun. 2019:10:1417. 30926845 10.1038/s41467-019-09344-1 PMC6441088

[ref40] Kiemes A, Davies C, Kempton MJ, Lukow PB, Bennallick C, Stone JM, Modinos G. GABA, glutamate and neural activity: a systematic review with meta-analysis of multimodal 1H-MRS-fMRI studies. Front Psychiatry. 2021:12:255. 10.3389/fpsyt.2021.644315. PMC7982484 33762983

[ref41] Kufahl PR, Li Z, Risinger RC, Rainey CJ, Wu G, Bloom AS, Li SJ. Neural responses to acute cocaine administration in the human brain detected by fMRI. NeuroImage. 2005:28:904–914. 16061398 10.1016/j.neuroimage.2005.06.039

[ref42] Levar N, Van Doesum TJ, Denys D, Van Wingen GA. Anterior cingulate GABA and glutamate concentrations are associated with resting-state network connectivity. Sci Rep. 2019:9:2116. 30765822 10.1038/s41598-018-38078-1 PMC6375948

[ref43] Li M, Danyeli LV, Colic L, Wagner G, Smesny S, Chand T, Di X, Biswal BB, Kaufmann J, Reichenbach JR, et al. The differential association between local neurotransmitter levels and whole-brain resting-state functional connectivity in two distinct cingulate cortex subregions. Hum Brain Mapp. 2022:43:2833–2844. 35234321 10.1002/hbm.25819 PMC9120566

[ref44] Liakoni E, Dempsey DA, Meyers M, Murphy NG, Fiorentino D, Havel C, Haller C, Benowitz NL. Effect of gamma-hydroxybutyrate (GHB) on driving as measured by a driving simulator. Psychopharmacology. 2018:235:3223–3232. 30232528 10.1007/s00213-018-5025-2 PMC6457903

[ref45] Liang P, Zhang H, Xu Y, Jia W, Zang Y, Li K. Disruption of cortical integration during midazolam-induced light sedation. Hum Brain Mapp. 2015:36:4247–4261. 26314702 10.1002/hbm.22914 PMC5049658

[ref46] Liechti ME, Quednow BB, Liakoni E, Dornbierer D, von Rotz R, Gachet MS, Gertsch J, Seifritz E, Bosch OG. Pharmacokinetics and pharmacodynamics of gamma-hydroxybutyrate in healthy subjects. Br J Clin Pharmacol. 2016:81:980–988. 26659543 10.1111/bcp.12863 PMC4834605

[ref47] Logothetis NK . What we can do and what we cannot do with fMRI. Nature. 2008:453:869–878. 18548064 10.1038/nature06976

[ref48] Magistretti PJ, Pellerin L. Cellular mechanisms of brain energy metabolism and their relevance to functional brain imaging. Philos Trans R Soc Lond Ser B Biol Sci. 1999:354:1155–1163. 10466143 10.1098/rstb.1999.0471 PMC1692634

[ref49] Mak LE, Minuzzi L, MacQueen G, Hall G, Kennedy SH, Milev R. The default mode network in healthy individuals: a systematic review and meta-analysis. Brain Connect. 2017:7:25–33. 27917679 10.1089/brain.2016.0438

[ref50] McCutcheon RA, Nour MM, Dahoun T, Jauhar S, Pepper F, Expert P, Veronese M, Adams RA, Turkheimer F, Mehta MA, et al. Mesolimbic dopamine function is related to salience network connectivity: an integrative positron emission tomography and magnetic resonance study. Biol Psychiatry. 2019:85:368–378. 30389131 10.1016/j.biopsych.2018.09.010 PMC6360933

[ref51] Menon V . Large-scale brain networks and psychopathology: a unifying triple network model. Trends Cogn Sci. 2011:15:483–506. 21908230 10.1016/j.tics.2011.08.003

[ref52] Menon V . Salience network. In: Toga AW, editors. Brain mapping: an encyclopedic reference. Waltham: Academic Press; 2015. pp. 597–611.

[ref53] Menon V, Uddin LQ. Saliency, switching, attention and control: a network model of insula function. Brain Struct Funct. 2010:214:655–667. 20512370 10.1007/s00429-010-0262-0 PMC2899886

[ref54] Overbeek G, Gawne TJ, Reid MA, Kraguljac NV, Lahti AC. A multimodal neuroimaging study investigating resting-state connectivity, glutamate and GABA at 7 T in first-episode psychosis. J Psychiatry Neurosci. 2021:46:E702–E710. 34933941 10.1503/jpn.210107 PMC8695527

[ref55] Pannekoek JN, Van Der Werff SJA, Meens PHF, Van Den Bulk BG, Jolles DD, Veer IM, Van Lang NDJ, Rombouts SARB, Van Der Wee NJA, Vermeiren RRJM. Aberrant resting-state functional connectivity in limbic and salience networks in treatment-naïve clinically depressed adolescents. J Child Psychol Psychiatry. 2014:55:1317–1327. 24828372 10.1111/jcpp.12266

[ref56] Pietrzykowski MO, Daigle KM, Waters AB, Swenson LP, Gansler DA. The central executive network and executive function in healthy and persons with schizophrenia groups: a meta-analysis of structural and functional MRI. Brain Imaging Behav. 2022:16:1451–1464. 34775552 10.1007/s11682-021-00589-3

[ref57] Plazzi G, Pizza F, Vandi S, Arico D, Bruni O, Dauvilliers Y, Ferri R. Impact of acute administration of sodium oxybate on nocturnal sleep polysomnography and on multiple sleep latency test in narcolepsy with cataplexy. Sleep Med. 2014:15:1046–1054. 25087195 10.1016/j.sleep.2014.04.020

[ref58] Power JD, Barnes KA, Snyder AZ, Schlaggar BL, Petersen SE. Spurious but systematic correlations in functional connectivity MRI networks arise from subject motion. NeuroImage. 2012:59:2142–2154. 22019881 10.1016/j.neuroimage.2011.10.018 PMC3254728

[ref59] Raichle ME, Mintun MA. Brain work and brain imaging. Annu Rev Neurosci. 2006:29:449–476. 16776593 10.1146/annurev.neuro.29.051605.112819

[ref60] Robinson DM, Keating GM. Sodium Oxybate. CNS Drugs. 2007:21:337–354. 17381187 10.2165/00023210-200721040-00007

[ref61] von Rotz R, Kometer M, Dornbierer D, Gertsch J, Salome Gachet M, Vollenweider FX, Seifritz E, Bosch OG, Quednow BB. Neuronal oscillations and synchronicity associated with gamma-hydroxybutyrate during resting-state in healthy male volunteers. Psychopharmacology. 2017:234:1957–1968. 28429067 10.1007/s00213-017-4603-z

[ref62] Scharf MB, Baumann M, Berkowitz DV. The effects of sodium oxybate on clinical symptoms and sleep patterns in patients with fibromyalgia. J Rheumatol. 2003:30:1070–1074. 12734908

[ref63] Shirer WR, Jiang H, Price CM, Ng B, Greicius MD. Optimization of rs-fMRI pre-processing for enhanced signal-noise separation, test-retest reliability, and group discrimination. NeuroImage. 2015:117:67–79. 25987368 10.1016/j.neuroimage.2015.05.015

[ref64] Studerus E, Gamma A, Vollenweider FX. Psychometric evaluation of the altered states of consciousness rating scale (OAV). PLoS One. 2010:5:e12412. 20824211 10.1371/journal.pone.0012412 PMC2930851

[ref65] Wang GY, van Eijk J, Demirakca T, Sack M, Krause-Utz A, Cackowski S, Schmahl C, Ende G. ACC GABA levels are associated with functional activation and connectivity in the fronto-striatal network during interference inhibition in patients with borderline personality disorder. NeuroImage. 2017:147:164–174. 27940074 10.1016/j.neuroimage.2016.12.013

[ref66] Wang J, Xu Y, Deshpande G, Li K, Sun P, Liang P. The effect of light sedation with midazolam on functional connectivity of the dorsal attention network. Brain Sci. 2021:11:1107. 10.3390/brainsci11081107 PMC8392174 34439725

[ref67] Xiao F, Lu C, Zhao D, Zou Q, Xu L, Li J, Zhang J, Han F. Independent component analysis and graph theoretical analysis in patients with narcolepsy. Neurosci Bull. 2019:35:743–755. 30421271 10.1007/s12264-018-0307-6 PMC6616568

[ref68] Yoo JH, Kim D, Choi J, Jeong B. Treatment effect of methylphenidate on intrinsic functional brain network in medication-naive ADHD children: a multivariate analysis. Brain Imaging Behav. 2018:12:518–531. 28417219 10.1007/s11682-017-9713-z

[ref69] Zhou H-X, Chen X, Shen Y-Q, Li L, Chen N-X, Zhu Z-C, Castellanos FX, Yan C-G. Rumination and the default mode network: meta-analysis of brain imaging studies and implications for depression. NeuroImage. 2020:206:116287. 31655111 10.1016/j.neuroimage.2019.116287

