# Supplementary material for: Subacute changes in brain functional network connectivity after nocturnal sodium oxybate intake are associated with anterior cingulate GABA
Source: Cereb Cortex. 2023 Mar 25;33(12):8046–55. doi: 10.1093/cercor/bhad097 (PMC10267648; doi:10.1093/cercor/bhad097)
Supplement: Bavato_et_al_2023_Suplemetary_materials_final_bhad097 [file bavato_et_al_2023_suplemetary_materials_final_bhad097.zip › Bavato_et_al_2023_Suplemetary_materials_final_bhad097.docx]

# **Supplementary Materials - Methods**

***MRS data acquisition.*** As reported in a separate publication from the same experiment (1), the ^1^H-MRS measurements started between 08:30-09:00 on a Philips Achieva 3T whole-body MR-unit using a 32-channel head coil (Philips Medical Systems, Best, The Netherlands). We used a two-dimensional J-resolved spectroscopy combined with PRESS localization (2D-JPRESS) sequence for spectroscopic acquisition 14. The 2D-JPRESS measures the signal as a function of the chemical shift, as well as of the coupling constant J, resulting in a 2D spectrum. The method allows the resolution of overlapping signals and enables the simultaneous detection of up to 20 metabolites including glutamate, glutamine and GABA (Supplementary Figure 1). We investigated a voxel with a nominal size of 38.2 cm3 (28 mm [L-R] x 31 mm [I-S] x 44 mm [A-P]) that was reduced to an effective voxel size of 14.8 cm3, due to the use of selective saturation pulses to render the data collection consistent for all metabolites of interest (denoted as OVERPRESS). As described before 15, we started with a minimal echo time (TE) of 30 ms that was incremented in 100 steps of 2 ms. We set the repetition time (TR) at 1600 ms and acquired eight averages for each TE-step. A qualified physicist used ProFit to quantify the metabolites as ratios to the total creatine 16. The data of four participants needed to be excluded from the MRS analyses because of excessive movements, technical issues with the MRS scanner or claustrophobia-induced interruption of scanning.

***Supplementary Figure 1:*** *Voxel placement and representative MR spectrum. (****A****) Voxel position in the bilateral anterior cingulate cortex on the anatomical scan (T1 weighted) of a representative subject. The blue slabs indicate the area suppressed by the saturation pulses. The red rectangle indicates the effective voxel and the green rectangle outlines the shim volume. (****B****) Two-dimensional JPRESS spectrum with the measured spectrum on top, the fitted spectrum in the middle, and the residuals (measured spectrum – fitted spectrum) at the bottom. (****C****) Cross-sectional view of the 2D-JPRESS spectrum shown in panel B along F1 = 0. The blue line shows the measured spectrum, the red line the fitted spectrum and the green line the residuum. Cho = choline; Cre = creatine; Glu = Glutamate; NAA = N-acetylaspartate. Figure obtained by Dornbierer et al. (1).*

***MRI data preprocessing.*** After 3D rigid body correction, a 24-parameter model of head motion was created for each data set with the purpose of regressing out any residual effects of motion from each data set prior to FC estimation. This model incorporates the 6 head motion translation and rotation parameters estimated during 3D rigid body correction, the 6 first order derivatives of the motion parameters, and the 12 corresponding squared items (2). Structural and functional data were co-registered and spatially normalized to the Talairach standard space using a twelve parameter affine transformation. In the course of this procedure, the functional images were resampled to an isometric 3 × 3 x 3 mm^3^ grid covering the entire Talairach box. Nuisance physiological signals from white matter (WM) and cerebro-spinal fluid (CSF) were estimated from each data set by segmenting the WM and the ventricles in the normalized T1 volumes and calculating the average WM and CSF signals from these volumes. Following previous recommendations (3, 4), all 24 motion parameters, together with the WM and CSF signals, were regressed out from each time-course at each voxel.

***Independent component analysis******.*** To select ICA components, we applied the RSN component templates derived from the baseline scans of a previously published dataset (acquired with the same parameters and the same MRI scanner) (5). We selected the DMN, the SN, and both the left and right CEN (l-CEN, r-CEN) as RSNs of interest and each RSN template was used to select one best-fitting RSN component per subject and per scan for the second-level random-effects statistical analysis (5, 6). The RSN with the highest goodness-of-fit score was chosen as the best-fitting component, defined as the difference between the average component score inside the mask minus the average component score outside the mask (6, 7). For each RSN of interest, all selected ICA component maps were entered into the second-level (group-level) statistical analysis. Besides the within-network connectivity changes within a given RSN of interest, the between-network connectivity changes (i.e. between two RSNs) were also assessed from the ICA component time-courses. Thus, the scan- and subject-specific ICA component time-courses were extracted for all individual ICA components selected as SN, DMN, r-CEN and l-CEN and correlation coefficients (Pearson's r) were computed per scan per subject for all possible pairs of ICA component time-courses for both individual scans. The resulting connectivity matrix was used for a second-level (group-level) statistical analysis after Fisher transformation from r to z values. Thereby, paired t-tests were conducted to compare correlation z values between different experimental conditions (GHB vs. placebo). Mean connectivity regression of the within-subject connectivity matrices was also performed, albeit without pre-whitening due to the choice of low-pass filtering the original time-courses (8, 9).

# **Supplementary Materials – Results**


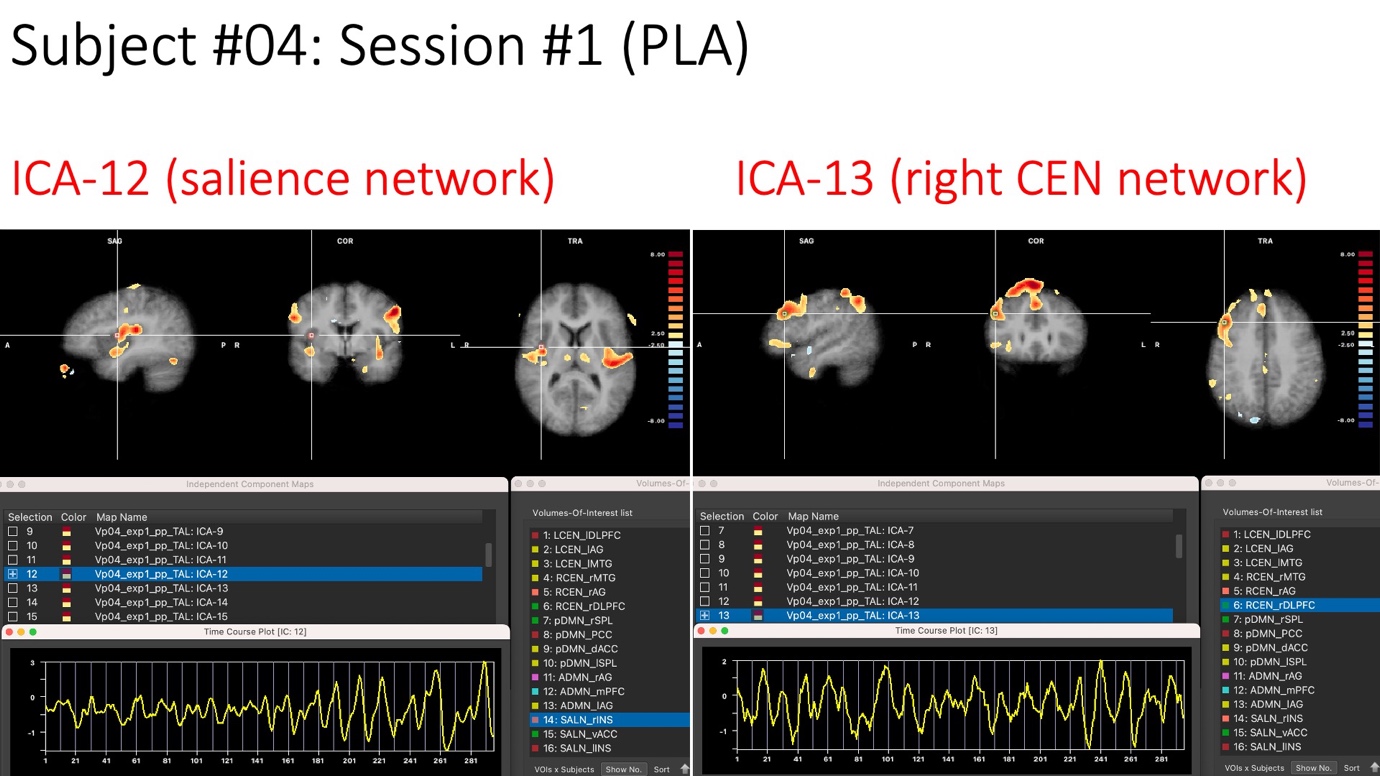


***Supplementary Figure 2.*** *Spatial distribution and functional connectivity time-course of salience and right central executive networks at placebo condition for subject #4.*


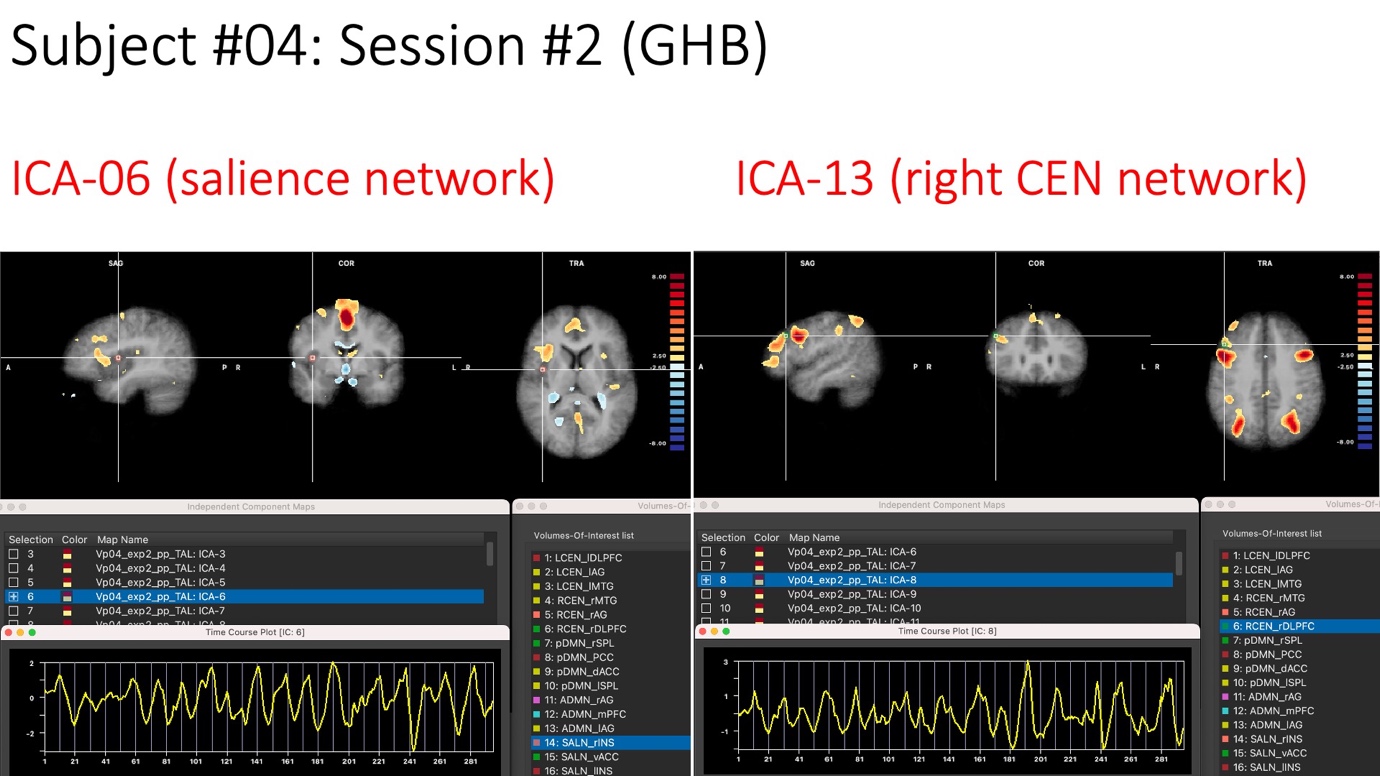


***Supplementary Figure 3.*** *Spatial distribution and functional connectivity time-course of salience and right central executive networks at GHB condition for subject #4.*


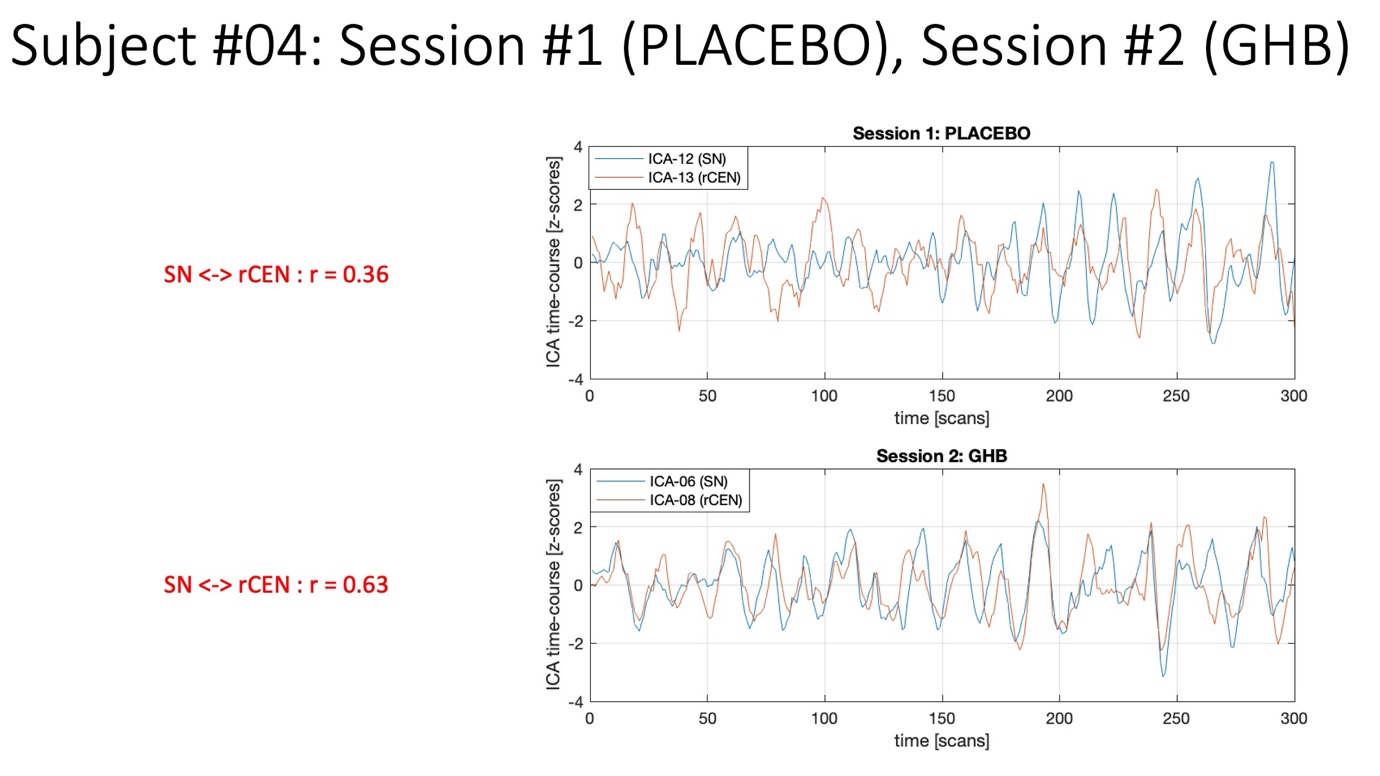


***Supplementary Figure 4.*** *Time-course of internetwork coupling between the salience and right central executive network at both condition for subject #4.*


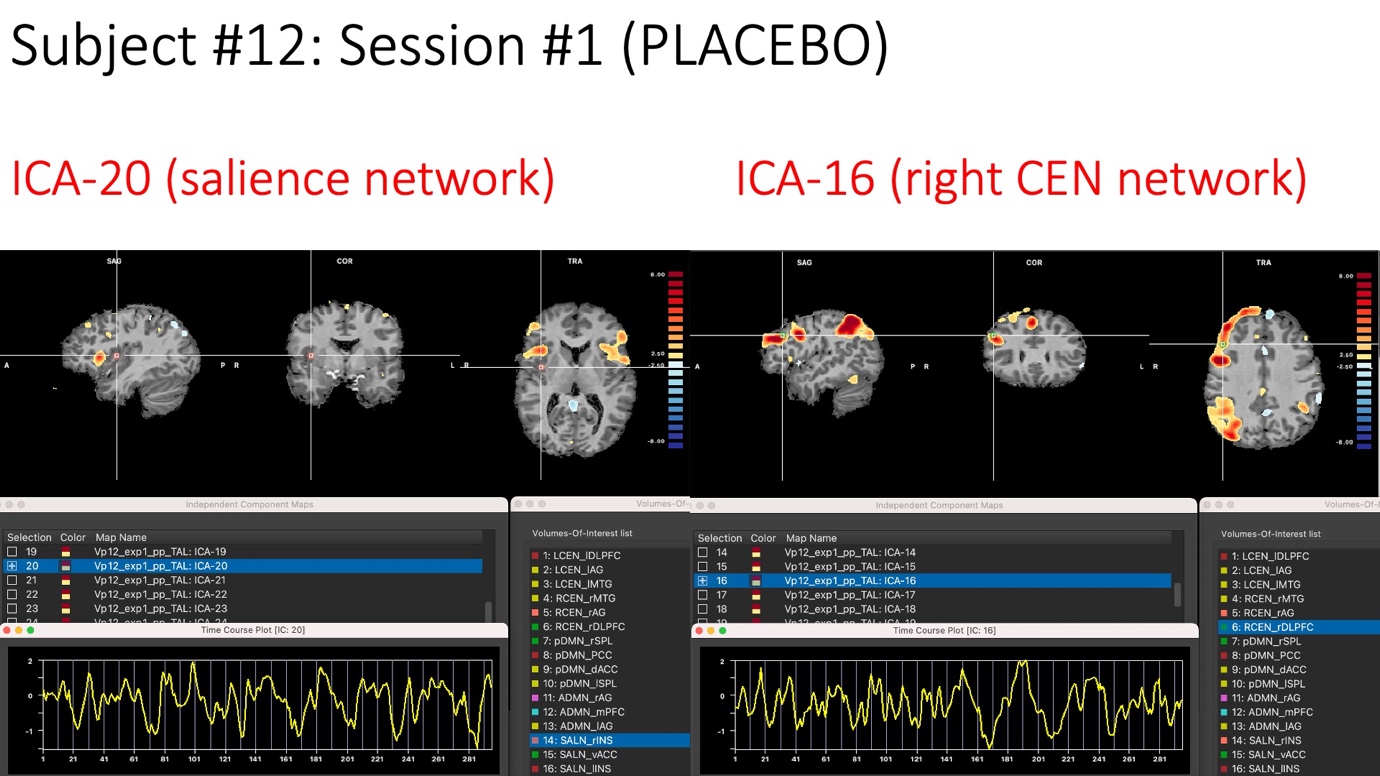


***Supplementary Figure 5.*** *Spatial distribution and functional connectivity time-course of salience and right central executive networks at placebo condition for subject #12.*


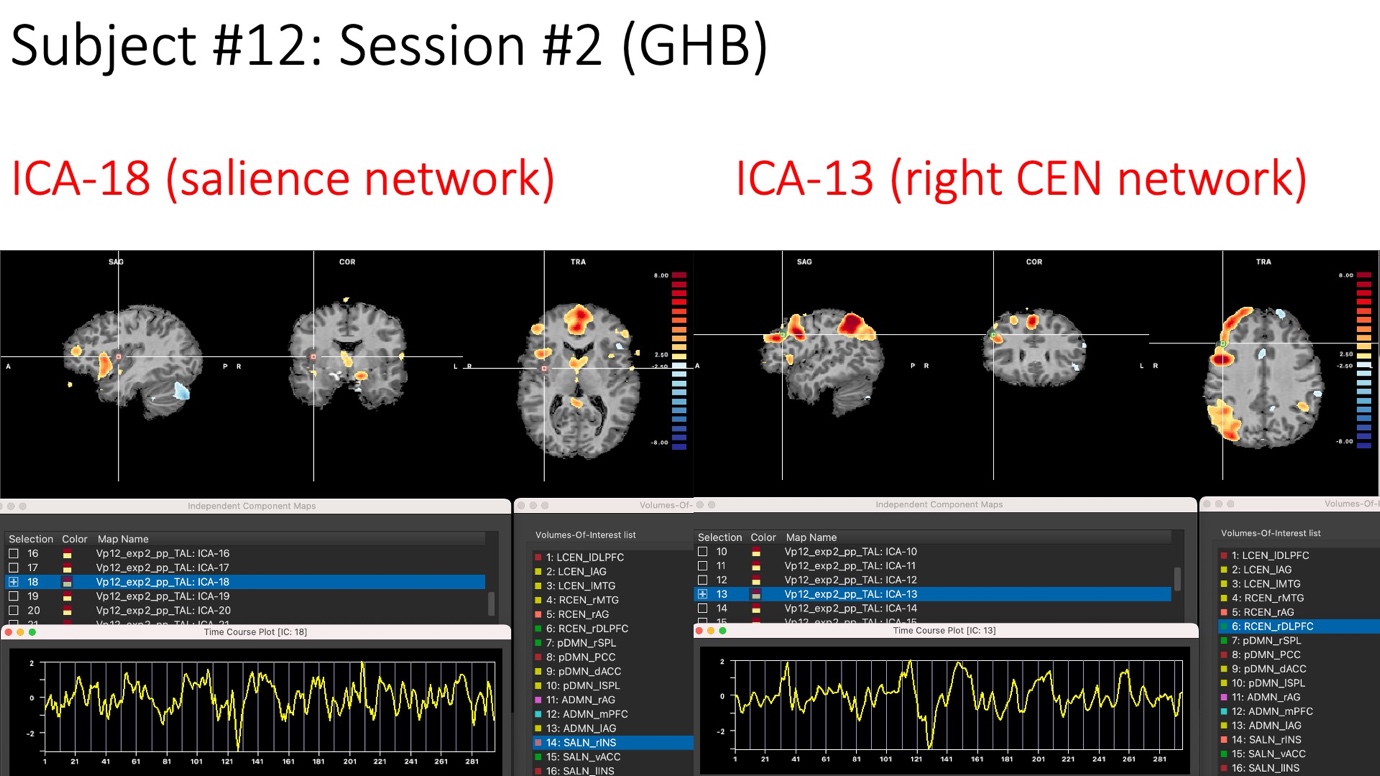


***Supplementary Figure 6.*** *Spatial distribution and functional connectivity time-course of salience and right central executive networks at GHB condition for subject #12.*


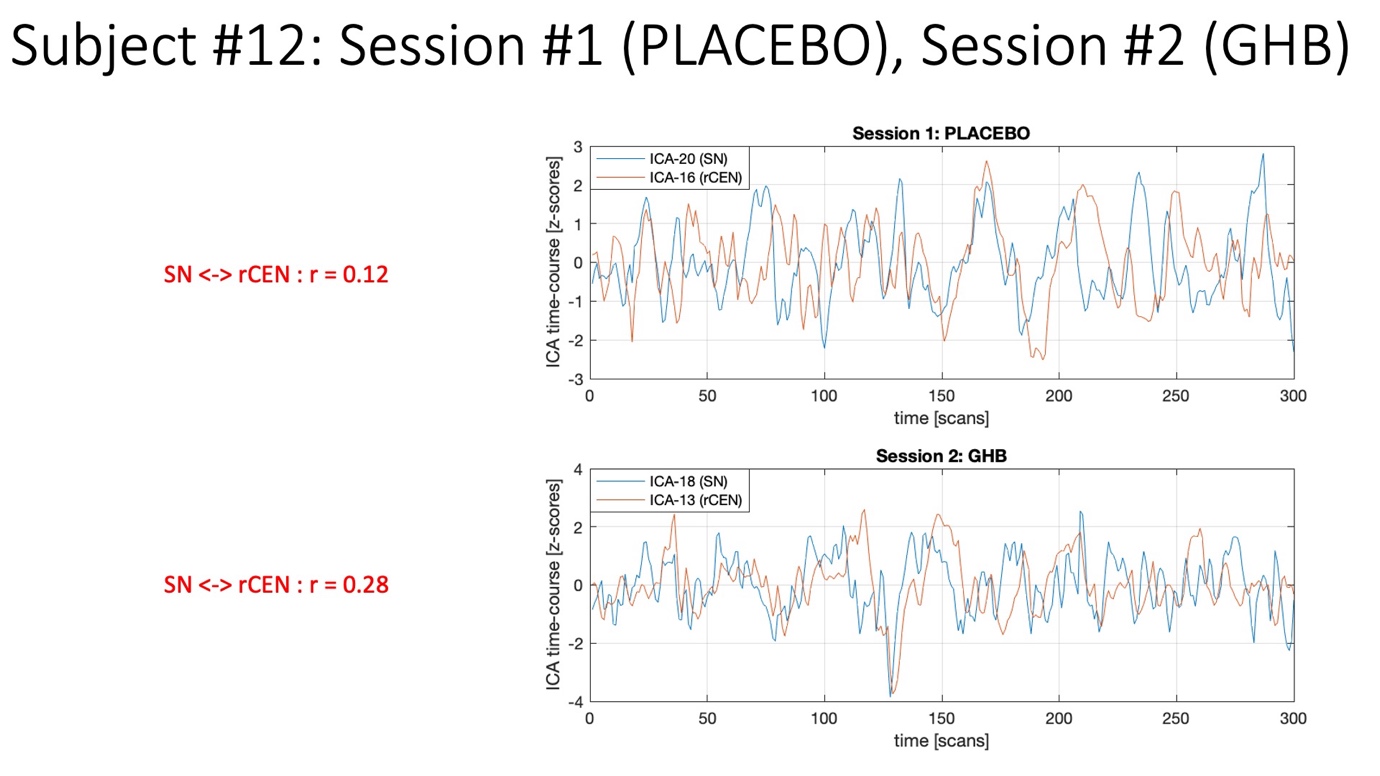


***Supplementary Figure 7.*** *Time-course of internetwork coupling between the salience and right central executive network at both condition for subject #12.*

**Supplementary Materials – Tables**

| **Supplementary Table 1. Subjective drug effects.** | | | | | | |
| --- | --- | --- | --- | --- | --- | --- |
| **EWL subscales** | **Mean ± SD Placebo** | **Mean ± SD**  **GHB** | ***Coefficient B*** | ***Standard error*** | ***Wald χ2*** | ***P value*** |
| Performance-related activation | 19.1 ± 4.4 | 19.1 ± 4.8 | 0.07 | 1.59 | 0.002 | 0.96 |
| General inactivation | 15.8 ± 3.7 | 16.8 ± 6.3 | -1.03 | 1.76 | 0.34 | 0.56 |
| Extro-/introversion | 19.8 ± 2.9 | 19.6 ± 3.6 | 0.19 | 1.12 | 0.29 | 0.87 |
| General well-being | 21.3 ± 5.1 | 20.0 ± 6.0 | 1.38 | 1.91 | 0.52 | 0.47 |
| Emotional sensitivity | 15.4 ± 3.5 | 16.0 ± 4.2 | -0.71 | 1.32 | 0.29 | 0.59 |
| Depressiveness/anxiety | 8.4 ± 0.7 | 9.1 ± 2.0 | -0.77 | 0.52 | 2.22 | 0.14 |
|  | | | | | | |
| *Generalized linear model with normal distribution and identity function. Dependent variable: EWL subscores; factor: treatment (placebo/GHB); co-variable: treatment order. Abbreviations: EWL: Eigenschaftswörterliste; SD: standard deviation.* | | | | | | |

**Supplementary Materials – Literature**

1. Dornbierer DA, Zölch N, Baur DM, Hock A, Stucky B, Quednow BB, Kraemer T, Seifritz E, Bosch OG, Landolt H-P. Nocturnal sodium oxybate increases morning anterior cingulate glutamate signal. Accepted, Journal of Sleep Research. 2023.

2. Satterthwaite TD, Elliott MA, Gerraty RT, Ruparel K, Loughead J, Calkins ME, Eickhoff SB, Hakonarson H, Gur RC, Gur RE, Wolf DH. An improved framework for confound regression and filtering for control of motion artifact in the preprocessing of resting-state functional connectivity data. Neuroimage. 2013;64:240-256.

3. Shirer WR, Jiang H, Price CM, Ng B, Greicius MD. Optimization of rs-fMRI Pre-processing for Enhanced Signal-Noise Separation, Test-Retest Reliability, and Group Discrimination. Neuroimage. 2015;117:67-79.

4. Varikuti DP, Hoffstaedter F, Genon S, Schwender H, Reid AT, Eickhoff SB. Resting-state test-retest reliability of a priori defined canonical networks over different preprocessing steps. Brain Struct Funct. 2017;222:1447-1468.

5. Bosch OG, Esposito F, Dornbierer D, Havranek MM, von Rotz R, Kometer M, Staempfli P, Quednow BB, Seifritz E. Gamma-hydroxybutyrate increases brain resting-state functional connectivity of the salience network and dorsal nexus in humans. Neuroimage. 2018;173:448-459.

6. Esposito F, Scarabino T, Hyvarinen A, Himberg J, Formisano E, Comani S, Tedeschi G, Goebel R, Seifritz E, Di Salle F. Independent component analysis of fMRI group studies by self-organizing clustering. NeuroImage. 2005;25:193-205.

7. Greicius MD, Srivastava G, Reiss AL, Menon V. Default-mode network activity distinguishes Alzheimer's disease from healthy aging: evidence from functional MRI. Proceedings of the National Academy of Sciences. 2004;101:4637-4642.

8. Yan CG, Cheung B, Kelly C, Colcombe S, Craddock RC, Di Martino A, Li Q, Zuo XN, Castellanos FX, Milham MP. A comprehensive assessment of regional variation in the impact of head micromovements on functional connectomics. Neuroimage. 2013;76:183-201.

9. Geerligs L, Tsvetanov KA, Cam C, Henson RN. Challenges in measuring individual differences in functional connectivity using fMRI: The case of healthy aging. Hum Brain Mapp. 2017;38:4125-4156.
